# Lipid-induced S-palmitoylation as a Vital Regulator of Cell Signaling and Disease Development

**DOI:** 10.7150/ijbs.64046

**Published:** 2021-10-11

**Authors:** Mengyuan Qu, Xuan Zhou, Xiaotong Wang, Honggang Li

**Affiliations:** 1 Institute of Reproductive Health/Center of Reproductive Medicine, Tongji Medical College, Huazhong University of Science and Technology, Wuhan, China.; 2 National Clinical Research Center for Infectious Disease; Department of liver Diseases, Shenzhen Third People's Hospital, Shenzhen, China.; 3 Wuhan Tongji Reproductive Medicine Hospital, Wuhan, China.

**Keywords:** Palmitoylation, Lipid metabolism, Cancer, Inflammation, Neurodegeneration

## Abstract

Lipid metabolites are emerging as pivotal regulators of protein function and cell signaling. The availability of intracellular fatty acid is tightly regulated by glycolipid metabolism and may affect human body through many biological mechanisms. Recent studies have demonstrated palmitate, either from exogenous fatty acid uptake or *de novo* fatty acid synthesis, may serve as the substrate for protein palmitoylation and regulate protein function via palmitoylation. Palmitoylation, the most-studied protein lipidation, encompasses the reversible covalent attachment of palmitate moieties to protein cysteine residues. It controls various cellular physiological processes and alters protein stability, conformation, localization, membrane association and interaction with other effectors. Dysregulation of palmitoylation has been implicated in a plethora of diseases, such as metabolic syndrome, cancers, neurological disorders and infections. Accordingly, it could be one of the molecular mechanisms underlying the impact of palmitate metabolite on cellular homeostasis and human diseases. Herein, we explore the relationship between lipid metabolites and the regulation of protein function through palmitoylation. We review the current progress made on the putative role of palmitate in altering the palmitoylation of key proteins and thus contributing to the pathogenesis of various diseases, among which we focus on metabolic disorders, cancers, inflammation and infections, neurodegenerative diseases. We also highlight the opportunities and new therapeutics to target palmitoylation in disease development.

## Introduction

Lipids are physiologically active and multifunctional, not only as energy supplies and nutritional components, but also play a vital role in cellular homeostasis maintenance, membrane organization, signal transduction, protein function and other biochemical reactions 1, 2. Recent studies showed the inextricable links between fatty acid (FA) biosynthesis, metabolic reprogramming and disease progression 3. Disruption of lipid metabolism has been involved in various pathological conditions, such as metabolic disorders, atherosclerosis, cancer, neurodegeneration, infections and immune diseases 4-6.

Palmitic acid (PA), a saturated fatty acid (SFA), is an important constituent of a typical westernized diet. It is universally found in human body and food, e.g. palm oil, meat, dairy products, cocoa butter 7. PA is also synthesized endogenously from other metabolites in a process known as *de novo* lipogenesis (DNL) 8, 9. And it could be transformed into phospholipids and sphingolipids as membrane components, utilized for β-oxidation as energy sources, or esterified to triacylglycerols for lipids storage. Apart from that, FAs provide substrates for protein lipidation, a group of co- or post-translational modifications including palmitoylation, myristoylation, prenylation, geranylgeranylation 10. Particularly in our case, PA is converted to palmitoyl-CoA and then incorporates into cysteine residues of protein via labile thioester linkage in a reversible process known as S-palmitoylation.

Proteins undergo palmitoylation and de-palmitoylation catalyzed by specific enzymes in response to upstream signals, which enable it to act as a regulator or a switch of protein function in a manner similar to protein ubiquitination or phosphorylation 11. Palmitoylation increases the hydrophobicity of proteins and plays a crucial role in regulating protein stability and function, localization and interaction, signal transduction 12. Dysregulation of palmitoylation has been implicated in a plethora of diseases (e.g. neurological diseases, cancers, metabolic syndrome, infections) 13-15, resembling the consequences of lipid homeostasis disruption.

Numerous inspiring progresses have been made recently, we consider it is essential to understand the cell signaling and molecular mechanism underlying the lipid-induced dysfunctions as well as the pathological relevance to palmitoylaiton, which may ultimately lead to novel therapeutics. Here, we focus on the elusive correlation between lipid metabolism and protein palmitoylation in different diseases. First, we will elucidate how metabolite palmitate, either from exogenous FA uptake or from *de novo* FA synthesis, regulates protein palmitoylation. Then, we review the updated evidences and uncover the potential mechanism by which lipid-related palmitoylation modulates protein function regarding disease development. Furthermore, we attempt to explore the opportunities and new strategies to target palmitoylation for therapeutic applications.

## Dynamic regulation and biochemical function of S-palmitoylation

S-palmitoylation can be regulated enzymatically and non-enzymatically. The dynamic cycle of palmitoylation takes seconds to hours to modulate protein biological functions 16. There are other types of palmitoylation, i.e. very few proteins with palmitate attached to serine residues (O-palmitoylation) or the N-terminus (N-palmitoylation) 17, 18. Other well studied protein lipidations, like myristoylation which involves the addition of a 14-carbon saturated myristate to N-terminal glycine residues via an amide linkage 19, prenylation which requires the attachment of prenyl groups to a cysteine residue via a thioether bond, are all irreversible 20. They exhibit resemblance in their functions and in the following part we focus on the function and regulation of S-palmitoylation.

**Figure 1:**
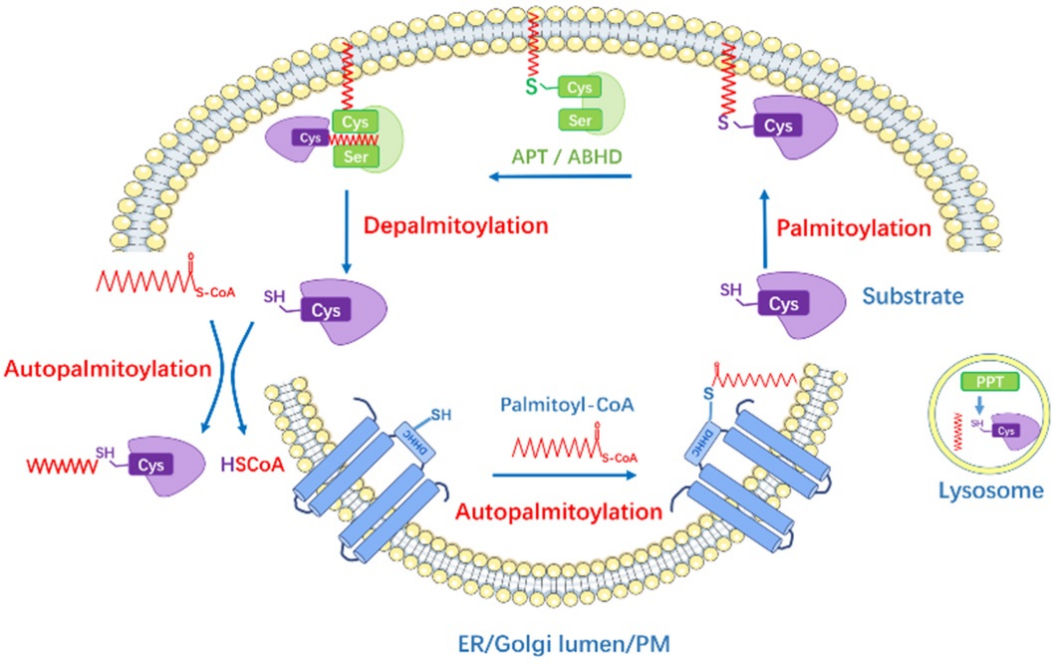
The Dynamic Regulation of Protein S-Palmitoylation. Palmitoyl acyltransferases perform the autopalmitoylation within the ZDHHC domain and then transfer palmitate to the substrate protein cysteines to accomplish palmitoylation. The depalmitoylation is catalyzed by thioesterases (APTs and ABHDs) which remove the palmitate from the proteins. PPTs are mainly localized in lysosome. Some proteins can be autopalmitoylation non-enzymatically. The lipid bilayers represent endoplasmic reticulum (ER), Golgi lumen and plasma membrane (PM).

S-palmitoylation is catalyzed by palmitoyl acyltransferases (PATs), also named zinc finger DHHC-type containing (ZDHHC) proteins because they possess a 51-amino acid zinc figure-like domain containing Asp-His-His-Cys (DHHC) motif 21. The conserved DHHC cysteine-rich domain represents the catalytic center and site-directed mutation of this motif impairs the palmitoyl-transfer ability. To date, 23 ZDHHC enzymes have been discovered in mammalian cells. Most ZDHHC proteins are localized in the Golgi apparatus and endoplasmic reticulum (ER), some in the plasma membrane while some in mitochondria 21-23. DHHC isoforms comprise of four to six transmembrane (TM) domains. The catalytic DHHC domain is located in the cytosolic part in between the second and third TM 24, 25. These domains target DHHC protein on different membrane and also form a binding pocket for Acyl-CoA. In contrast, the intracellular C- and N-terminal cytoplasmic tails possess great sequence diversity and mediate protein-protein interaction, thus bring the substrates closer and facilitate the acyl‐transfer process 24. Rana et al. 26 recently reported the crystal structure of DHHC20: the four TMs of zDHHC20 form a tepee-like cavity where the fatty acyl chain binds, and the residues determine the acyl CoA chain length selectivity. The ZDHHCs catalyze protein palmitoylation in a two-step process, which is autopalmitoylation of themselves to form an acyl-enzyme intermediate, and subsequently transfers the Acyl-CoA to the targeted cysteine residue in substrate protein** (Figure 1)**. Despite the similar zinc figure-like motif, ZDHHCs display distinct abilities to autoacylate, indicating they have different preferences in substrate proteins and uneven levels of catalytic efficiency 27, 28: palmitoylated proteins may respond to more than one ZDHHC enzymes and one ZDHHC can have multiple substrates. The regulatory mechanism underlying how ZDHHCs select candidates for modification and their functional redundancy are not entirely clear.

The enzymatic thioester hydrolysis that removes palmitate from palmitoylated proteins is catalyzed by a family of serine hydrolases, including acyl-protein thioesterases (APT1, APT2), palmitoyl protein thioesterases (PPT1, PPT2), the α/β hydrolase domain proteins (ABHD) 29-31 **(Figure 1)**. APTs were previously thought to reside in the cytosol and Golgi apparatus and depalmitoylate membrane-anchored proteins 32, yet recently, APT1 was found to mediate depalmitoylation in mitochondria 33. Meanwhile, Abrami et al. 34 has reported that APT2 membrane binding requires three steps: electrostatic attraction, a hydrophobic loop insertion and S-acylation by ZDHHC3 or ZDHHC7, afterwards deacylation of substrates and hydrolysis may take place. PPTs are mainly localized in lysosome to depalmitoylate proteins before lysosomal degradation 30, while PPT1 also exhibits multiple cellular localizations 35. ABHD17 proteins are the newly discovered enzymes that are able to depalmitoylate NRAS and PSD-95 efficiently, playing important roles in tumorigenesis and neuro-system, respectively 31, 36. However, whether other members in ABHD family have the same physiological functions is largely unknown.

In addition, autopalmitoylation usually means the spontaneous autoacylation of a specific protein in the presence of Acyl-CoA via zDHHC-independent pathway 37. Previously, autoacylation was thought to be a nonspecific reaction that happened when certain cysteine residues encountered high palmitoyl-CoA levels (like over 100μM) 38. However, studies of yeast Bet3 protein 39, myelin P0 glycoprotein 40 and transcriptional enhanced associate domain (TEAD) proteins 41 have demonstrated that autoacylation can occur in physiological status due to their special structure, some of them possess intrinsic “enyme-like” activities and bind to palmitoyl-CoA directly. In fact, only few specific proteins with strong Acyl-CoA binding ability could be autoacylated. Increased dose of palmitate treatment seems to enhance the autoacylation process *in vitro*, but the underlying mechanism is poorly understood.

Palmitoylated proteomes have identified thousands of palmitoylated proteins, the actual number is even larger. Many receptors, enzymes, kinases, transcription factors and oncogenes are palmitoylated, and palmitoylation of key signaling proteins, such as STING, RAS and Hedgehog, is essential for their pathophysiological functions 42, 43. For instance, NRAS signaling demands palmitoylation for its membrane localization and signaling transduction in leukemogenesis 44. Palmitoylation of G protein-coupled receptors (GPCRs) is important for targeting GPCRs to lipid rafts and affecting all aspects of GPCR signaling, such as the phosphorylation status, desensitization and internalization of the receptor 45. Ion channel can be regulated by palmitoylation as well. Long-chain Acyl-CoA esters, like palmitoyl-CoA, exert acute and direct regulation over K_ATP_ channels, and also mediate the regulation through palmitoylation 46. Increased palmtoylation of Kir6.2 at Cys^166^ when pre-incubated with micromolar concentrations of PA can promote the channel open state 46. Therefore, understanding the function of palmitoylation is crucial to comprehend the molecular mechanism and therapeutics of diseases.

## Fatty acids bio-availability as a key regulator of S-palmitoylation

**Figure 2:**
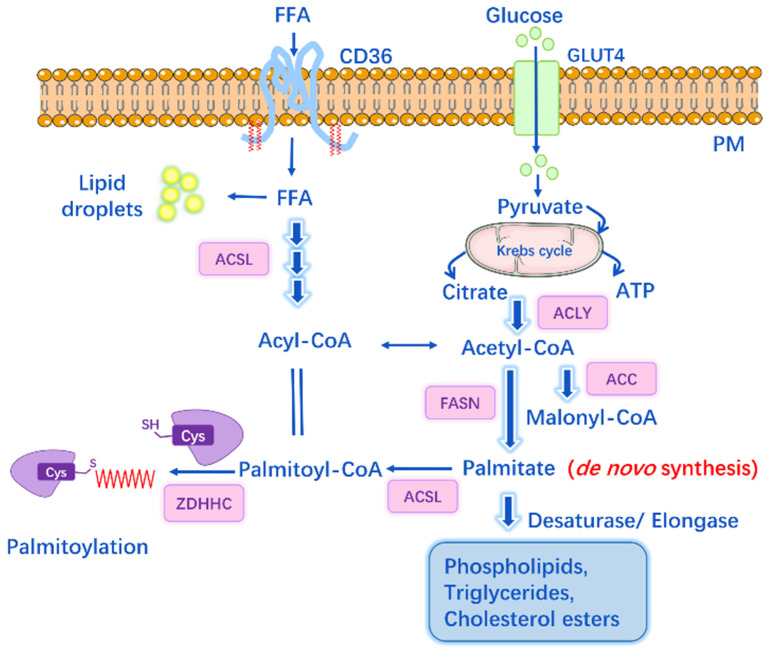
The link between lipid metabolism and S-palmitoylation. Glucose transporter (GLUT4) transports glucose into cell while CD36 mediates the uptake and endocytosis of exogenous free fatty acids (FFAs). Acetyl-CoA is generated from citrate by ATP-citrate lyase (ACLY); and then, Acetyl-CoA is carboxylated to malonyl-CoA by Acetyl-CoA carboxylase (ACC). Fatty acid synthase (FASN) is the key rate-limiting enzyme that catalyzes the synthesis of palmitate from Acetyl-CoA and malonyl-CoA (the byproducts of glucose metabolism and Krebs cycle). Long-chain Acyl-CoA syntheses (ACSL) catalyzes FAs into Acyl-CoAs, e.g. Palmitoyl-CoA, which is the substrate for palmitoylation.

S-palmitoylation can be influenced by factors other than PATs or APTs, such as *de novo* palmitate synthesis by fatty acid synthase (FASN) 47. It is well-known that under physiological conditions, intracellular concentrations of FAs and their derivatives are tightly regulated by cellular metabolism. Physiological palmitoyl-CoA concentration within cells ranges from 100 nM to 10 μM, depending on the tissue and metabolic state 48, and the acyl-CoA concentration can be a feedback regulation of FASN activity. Specifically, Acetyl-CoA is generated from citrate by ATP-citrate lyase, and then Acetyl-CoA is carboxylated to malonyl-CoA by Acetyl-CoA carboxylase (ACC). Subsequently, FASN catalyzes the synthesis of palmitate from Acetyl-CoA and Malonyl-CoA. Circulating palmitate is transported into cells and converted to palmitoyl-CoA, which can be the substrate for palmitoylation** (Figure 2)**. Overexpression of FASN facilitates the synthesis of endogenous palmitate and thus potentially increases palmitoylation, while inhibitors of FASN suppress the palmitoylation of certain proteins (e.g. EGFR, PD-L1, MYD88) and then affect protein stability, cellular localization, function and the interaction with other effectors 47, 49, 50.

Acyl-CoA synthetases (ACS) also play important roles in lipid metabolism by catalyzing FAs into Acyl-CoAs. Different ACS enzymes exhibit preferences for FA substrates of varying chain lengths, ranging from 2 to over 30 carbon atoms. Among them, long-chain Acyl-CoA synthetases (ACSL1-5), short-chain Acyl-CoA synthetases (ACSS) are of particular importance **(Figure 2)**. Pathophysiological conditions like cancer or inflammatory diseases may notably induce DNL, resulting in increased lipid levels and enhanced expression of FASN, ACSS, ACSL 49. For instance, ACSS2 is found overexpressed in cisplatin-resistant bladder cancer cells, while inhibition of ACSS2 can decrease FA synthesis by over 60% and affect the palmitoylation of specific proteins that regulate cell migration and proliferation 49. Studies have shown that ACSLs are positively associated with the palmitoylation levels of several proteins (e.g. Wnt2B, eNOS, Gs alpha), and blockade of ACSLs by triacsin C results in inhibited palmitoylation 51-53.

CD36 is another key player in lipid homeostasis by mediating the uptake and intracellular trafficking of exogenous FAs 54. It can also affect AMPK pathway and modulate FA β-oxidation 55. The expression of CD36 can be upregulated by hyperglycemia and hyperlipidemia. CD36 accounts for 50% of the FA uptake in adipose tissues and muscle in mice, hence CD36 deficient mice display decreased FA uptake and elevated fasting levels of long-chain FAs, cholesterol and triacylglycerol 56. When treating cells with CD36 inhibitor SSO or cultured in delipidated fetal bovine serum, MYD88 palmitoylation slightly decreases 47. Thus, palmitoylation of certain proteins might be affected by CD36-mediated uptake of exogenous FAs.

PA might influence the autopalmitoylation process. Kim NG's research suggested that *de novo* palmitate synthesis affected the autopalmitoylation of TEAD, and the depletion of FASN showed dramatic suppressing effects on TEAD activity and stability 57. In addition, PA can be converted to stearic acid and vice versa. Therefore, stearic acid and stearoyl-CoA also represent an important source of FAs for protein lipid modifications. For instance, FAs affect TFR1 acylation levels and subsequent activation of JNK signaling in Drosophila 58. Transcription factor RFX3 can be autopalmitoylated in a palmitoyl-CoA concentration-dependent manner at a conversed cysteine residue without enzyme, however, it also exhibits preferences for 18-carbon FAs, and fatty acylation is required for RFX3 dimerization and transcriptional activity 59. Pharmacological inhibition of ACSL can suppress RFX3's acylation in cells, indicating that intracellular metabolite FAs might directly regulate protein autoacylation and signal transduction 59. Furthermore, ZDHHCs undergo autoacylation when incubated with palmitoyl-CoA. It is possible that high availability of palmitate contributes to the activation of ZDHHC by palmitoylating them, or causes ZDHHC overexpression, and then promote the palmitoylation of specific substrates 60. In particular cases, ZDHHC can autopalmitoylate and also be palmitoylated by another ZDHHC. ZDHHC16 palmitoylates ZDHHC6 at Cys^328^, Cys^329^, and Cys^343^ within the C-terminal tail, this palmitoylation “cascade” controls and enhances the overall stability and activity of ZDHHC6 61.

It is still uncertain how palmitoylation responses to the cellular FAs and metabolite signals in the pathogenesis of various diseases. It is possible that dietary intake lipids and cellular metabolites could affect the cellular palmitoyl-CoA levels, and in turn contribute to the alteration of protein palmitoylation. Aberrant palmitoylation may subsequently contribute to the pathological changes in diseases development. In the following sections, we will discuss it from four major aspects, including metabolic disorders, cancer development, inflammation and infection, neurodegeneration.

## Lipid-induced metabolic disorders via palmitoylation

**Figure 3:**
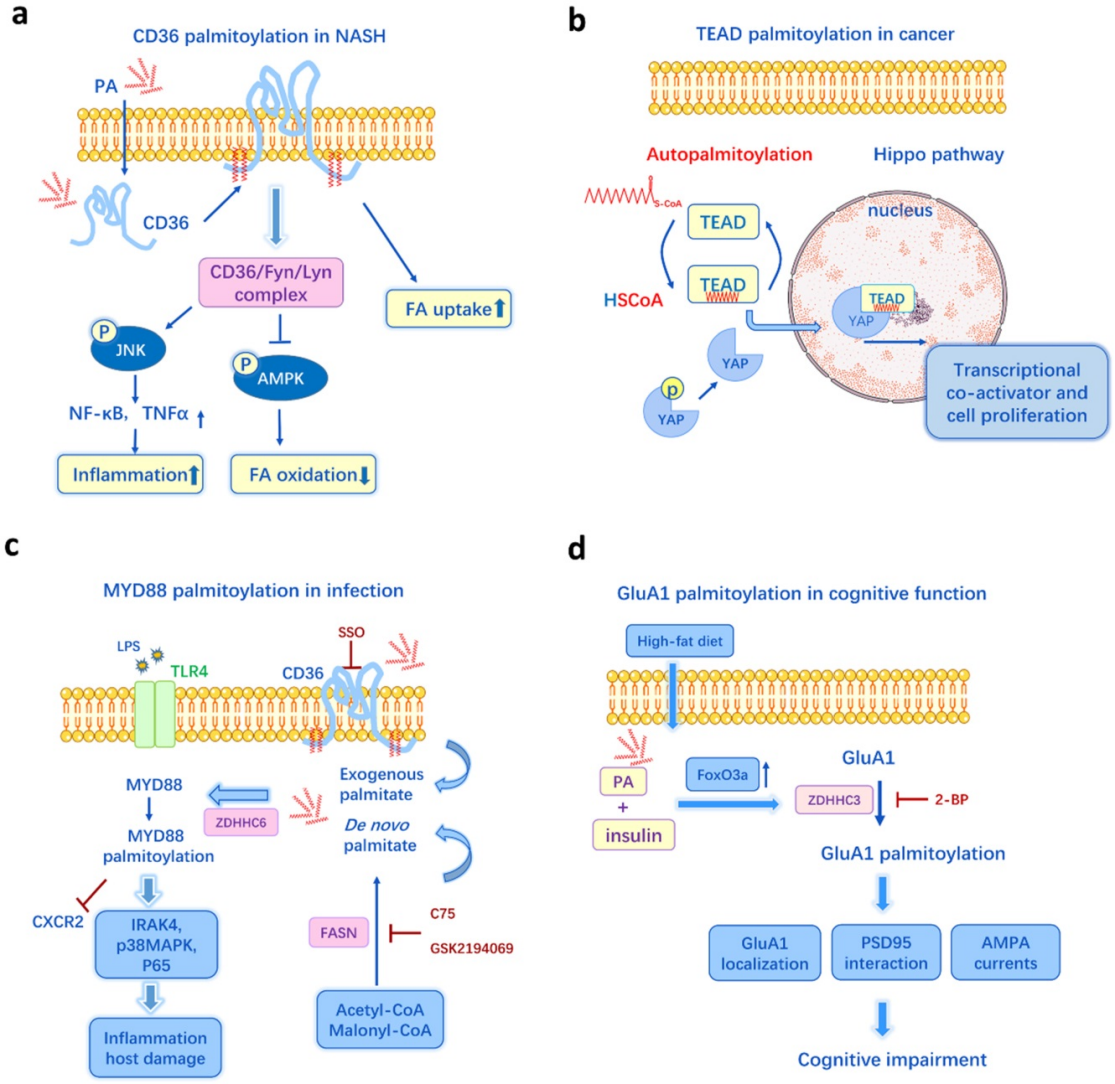
Schematic examples of protein palmitoylation and metabolism, cancer, inflammation, neurodegeneration. **a.** In the model of non-alcoholic steatohepatitis (NASH), palmitate induces CD36 palmitoylation, and the enhanced palmitoylation promotes the CD36/Lyn/Fyn complex, impairs FA β-oxidation, causes lipid accumulation, increases inflammation and cytokines release. **b.** Transcriptional enhanced associate domain (TEAD) is autopalmitoylated and it could be affected by intracellular palmitate levels. Palmitoylation of TEAD is required for its association with YAP and the regulation of transcriptional output of Hippo signaling. **c.** The *de novo* synthesis of palmitate by FASN and CD36-mediated exogenous FA uptake are two important sources for MYD88palmitoylation, which is essential for IRAK4 recruitment, p38MAPK and p65 activation, TLR4/MYD88 pathway. **d.** Palmitate deposition in the hippocampus increases GluA1 palmitoylation through FoxO3a-mediated overexpression of zDHHC3, resulting in altered GluA1 localization and function, suppressed AMPAR response and impairment of cognitive function. Figures include data from references 62, 41, 47, 60.

The oversupply of dietary fats and disruption of lipid metabolism can lead to numerous metabolic diseases, including obesity, diabetes, atherosclerosis, cardiovascular diseases and non-alcoholic steatohepatitis (NASH). In a NASH mouse model, a high-fat diet (HFD) and PA treatment can induce liver CD36 palmitoylation dose-dependently *in vivo* and *in vitro* 62. The enhanced palmitoylation of CD36 in NASH facilitates fatty acid uptake and impairs FA β-oxidation, causing lipid accumulation in liver. Inhibition of CD36 palmitoylation reduces FA binding/uptake and activates the AMPK pathway, thus ameliorates intracellular lipid accumulation and prevents NASH development 62** (Figure 3a)**. Meanwhile, DHHC4 and DHHC5 are two PATs required for the plasma membrane localization of CD36 and FA uptake activity in adipose tissues 63. And knockout of DHHC4 or DHHC5 in mice resembles the phenotype of CD36 deficient mice 63. Additionally, it has been recently identified that the key mechanism by which FAs are transported across the plasma membrane in adipocytes is CD36-mediated endocytosis and this endocytosis depends on the dynamic palmitoylation of CD36 64. Restricting CD36 at either palmitoylated or depalmitoylated status blocks the FA uptake activity 64. Since CD36 is a key player in metabolic diseases, like NASH, atherosclerosis 65, diabetes 66, and it can be influenced by both lipid levels and palmitoylation, CD36 palmitoylation is likely to be a crucial mechanism underlying lipid-induced metabolic disorders.

GLUT4 is another vital regulator in glucose transport and metabolic homeostasis, and GLUT4 dysfunction causes hyperglycemia and insulin resistance, leading to type 2 diabetes 67. The palmitoylation of GLUT4 plays a pivotal role in GLUT4 membrane translocation and glucose uptake 68. GLUT4 palmitoylation changes in a glucose and/or insulin-dependent manner. Insulin induces DHHC7 autopalmitoylation and activity, and then increases GLUT4 palmitoyaltion level, while inhibition of DHHC7 suppresses insulin-dependent GLUT4 translocation in adipocytes, subsequently disturbing the glycometabolism 69. This indicates palmitoylation is carefully regulated in an organism by the insulin and glycolipid metabolic homeostasis, while diets account for only a small part of the regulation. Moreover, other effectors in the insulin signaling like IRAP and SNARE complex (including Syntaxin4, SNAP23, and VAMP2) also require palmitoylation for glucose uptake 69-71. Interestingly, in the db/db mice, a model of type 2 diabetes with insulin resistance, the APT1 enzyme activity is identified to be impaired, leading to enhanced R-Ras palmitoylation, altered R-Ras trafficking, and thus defective vessel maturation in peripheral artery disease 72. Endothelial knockout of APT1 in mice has the similar phenotype to hyperglycemia stimulation 72. In this case, the aberrant palmitoylation in response to the metabolic milieu may represent an unrecognized mechanism underlying the metabolic vascular diseases.

As the key rate-limiting enzyme in DNL, FASN could affect the palmitoylation level of endothelial nitric oxide synthase (eNOS). It has been shown that the endogenously synthesized palmitate is the preferred source that palmitoylated eNOS 73. In FASN-deficient endothelial cells and insulin-resistant diabetic mouse model, eNOS palmitoylation is decreased, resulting in defective angiogenesis and endothelial dysfunction 73. Meanwhile, the activity of eNOS is important for FA dependent β-oxidation in muscle. PA can increase eNOS palmitoylation in left ventricular myocytes in healthy rats, whereas in hypertensive rats, PA has no effect on eNOS palmitoylation or myocyte contraction, so the relationship between PA and palmitoylation varies following specific situations 74.

FASN is an insulin responsive enzyme which also plays a role in maintaining the intestinal mucus barrier. A diabetic mouse model exhibits lower insulin level and decreased intestinal FASN expression. And FASN deficiency results in the inhibition of Mucin 2 palmitoylation, leading to the disruptions in the intestinal mucus barrier. Moreover, this could be restored with insulin treatment 75. FASN-dependent palmitoylation of Mucin 2 is essential for normal Muc2 secretion and function, however, FASN deficiency does not affect Claudin-1 palmitoylation, a member of well-known tight junction proteins, indicating FASN has selective effects on palmitoylation of different targets 75. Overall, the impaired insulin-FASN-Mucin2 palmitoylation axis explains the pathogenesis of intestinal inflammation in obese or diabetic patients, and also highlights palmitoylation as a link between gut lipogenesis and intestinal barrier function.

In a previous proteomics of adipocytes, over 800 putative palmitoylated proteins are identified, including proteins in JAK-STAT pathway and several kinases, like JAK1 and 2, STAT1, 3, 5A, ERK1/2 and AMPKα 70. Those are important proteins that may modulate signal transduction and energy metabolism in adipocytes. *Zdhhc13*-deficient mice exhibit abnormal liver function, lipid abnormalities, and hyper-metabolism 23. According to the S-palmitoylome, palmitoylation of 254 proteins is downregulated in *Zdhhc13*-deficient mouse liver, such as FASN, ACAA1, ACAA2 and CTNND1. These potential substrates of ZDHHC13 are essential for FA metabolism and β-oxidation, and palmitoylation may provide a membrane anchor for their translocation and maintain normal cellular function 23. Proteomics of human endothelial cells shows there are almost 10% of the putative palmitoylated proteins affected by insulin 76. For instance, PAFAH1b3 palmitoylation is upregulated by insulin exposure and required for endothelial function 76. The mechanism underlying insulin-related palmitoylation is unknown, but insulin can affect glycolipid metabolism and palmitate level, change enzyme activity as well 60, 72, all of which are the essentials for palmitoylation.

Over-nutrition like high glucose/palmitate leads to increased oxidative stress via palmitoylation. An increased autopalmitoylation of cytosolic thioredoxin reductase (TrxR1) and thioredoxin (hTrx1) occurs when incubated with high concentration of palmitoyl-CoA 77. However, enhanced TrxR1/hTrx1 palmitoylation causes an inhibition of their activities and an induction of oxidative stress in liver cells 77. This is consistent with the research that a HFD can induce TrxR1/hTrx1 palmitoylation and negatively impact these two critical antioxidant proteins in mouse liver 78. Moreover, palmitate induces ER stress in pancreatic β cells, resulting in β cell apoptosis and impaired insulin production in diabetes 79. Excess or aberrant palmitoylation could be one mechanism by which palmitate caused ER stress activation and β cell toxicity, since 2-Bromopalmitate (2-BP) or cerulenin, the inhibitor of palmitoylation, could largely attenuate the ER stress-mediated apoptosis 80. Another example is that incubation with palmitoyl-CoA (0.5-5 μM) causes concentration-dependent inhibition of GAPDH dehydrogenase activity via GAPDH palmitoylation at Cys^244^. Moreover, palmitoylated GAPDH contributes to the maladaptive changes in metabolic disorders 81. Palmitoylation has also been observed in mitochondria 82. Palmitate stimulation alters mitochondrial palmitoyl-CoA levels and results in increased rate of mitochondrial palmitoylation/depalmitoylation cycles 33. The fact mitochondrial palmitoylation can respond dynamically to local lipid levels is very intriguing, since mitochondria is the major organelle for β-oxidation and energy homeostasis.

However, other researches support that HFD-induced metabolic stress alters palmitoylation in a different direction. In mice fed with a high-fat, high-sucrose diet or in palmitate/glucose-stimulated endothelial cells, there was a reduction in HRas palmitoylation, leading to decreased ERK phosphorylation and apoptotic signaling activation 83. A similar study shows LIM domain only 4 (LMO4) palmitoylation is inhibited by a brief HFD or acute intracerebroventricular infusion of palmitate in mice, meanwhile, the inhibited palmitoylation retains LMO4 at the ER and increases protein tyrosine phosphatase 1B activity in the hypothalamus, resulting in leptin resistance 84. It is intriguing that dietary lipids have varied effects on the palmitoylation of different proteins. And it is improper to jump to conclusion that a high-fat/sucrose diet or obesity could increase the palmitoylation of a certain protein. This is a very complicated regulatory network and the mechanism by which HFD decreased the protein palmitoylation is unclear, but still, it is noteworthy that lipid-induced alteration of palmitoylation plays a pivotal part in the regulation of metabolism and the whole body hemostasis.

## Lipid metabolism and palmitoylation in cancer

Metabolic reprogramming in cancer cells enhances cell proliferation and survival 85. In normal cells, circulating lipids from dietary fats are preferentially chosen, excess FAs are converted to triacylglycerol for storage or β-oxidation when need energy, whereas tumors undergo exacerbated *de novo* FA synthesis by FASN, irrespective of the extracellular FA levels 86. The upregulation of FASN widely occurs in cancers and usually indicates cancer progression, metastasis, recurrence and chemo-resistance 86, 87. On the other hand, palmitoylation is frequently discovered in various cancers and constitutes one of the diverse molecular mechanisms in cancer development 15. To be specific, 26% of cancer driver gene encoded proteins that can be palmitoylated according to 15 palmitoylome studies 15, 88. Moreover, Dixon's team also summarized individual ZDHHC enzymes that act as either oncoproteins or tumor suppressors in a tissue-specific manner 15. So whether lipid metabolism affects cancer development through regulating the palmitoylation of cancer-related proteins is worth exploring.

Epidermal growth factor receptor (EGFR) is an oncogenic receptor tyrosine kinase associated with multiple human malignancies 89. The palmitoylation of EGFR is FASN-dependent and required for EGFR dimerization and kinase activation 90. Inhibition of FASN by cerulenin leads to downregulation of EGFR palmitoylation, and thus sensitize tumor to EGFR tyrosine kinase inhibitors in several cancer cell lines 90. In EGFR mutated non-small cell lung cancer acquired tyrosine kinase inhibitor resistance, the resistance relies on EGFR palmitoylation and FASN. Orlistat, an FDA-approved anti-obesity drug and also the inhibitor of FASN, blocks EGFR palmitoylation, alters EGFR cellular distribution, induces EGFR ubiquitination, and thus reduces tumorigenesis *in vivo* and *in vitro* 50. On the other hand, Runkle et al. 91 has demonstrated that targeting DHHC20 and inhibiting EGFR palmitoylation could increase sustained EGFR signal activation and sensitize tumor to EGFR inhibitor-induced cell death. And recently, they also found blocking EGFR palmitoylation inhibited activation of the kinase PI3K and reduced tumor growth in KRAS-mutant lung cancer 92. A rationale for the differences may be the mutational status of EGFR that affects the functional role of palmitoylation on EGFR, as the structural differences exist between wild-type and mutant EGFRs, causing different binding protein partners and different mechanisms.

Wnt signaling is another key oncogenic pathway in multiple cancers while Wnt proteins are modified by palmitoylation 93. Palmitoylation of Wnt/β-catenin pathway is identified as a potential mediator of FASN oncogenicity in the prostate cancer, that is, overexpression of FASN increases incorporation of *de novo* palmitate into Wnt-1, and enhanced Wnt-1 palmtoylation in prostate epithelial cells causes cytoplasmic β-catenin accumulation and pathway activation 94. In addition, FA synthesis may also induce invasion and metastasis via increased palmitoylation. FASN can modulate palmitoylation of RhoU, an atypical GTPase involved in actin cytoskeletal remodeling and cell migration 95. FASN depletion leads to decreased RhoU palmitoylation and impaired RhoU activity, and thus the down-regulation of paxillin serine phosphorylation and reduced migration phenotype in prostate cancer 95. On the other hand, it is noteworthy that the exogenous palmitate taken up by the cells can restore RhoU palmitoylation and activate invasion. In some case, cancer cells with high expression of CD36 and lipid metabolism genes show high metastatic potential 96, it is intriguing whether palmitoylation is involved and related to the high FA bio-availability.

Programmed death receptor ligand (PD-L1) is overexpressed in many types of cancer cells and causes tumor cells to escape from immune surveillance through binding to its receptor PD-1 on T cells 97. Previous studies suggests FASN expression is positively correlative with PD-L1 level in cisplatin-resistant lung cancer cells and a human T-cell leukemia line 98, 99, and FASN inhibitor orlistat could suppress cell proliferation and downregulate PD-L1 expression 99. A recent proteomics discovers that PD-L1 is FASN-dependent palmitoylation in bladder cancer cisplatin resistance and inhibition of FASN suppresses PD-L1 palmitoylation and expression 49. In fact, palmitoylation can stabilize PD-L1 and promote tumor growth in breast cancer 100, colorectal cancer, and other types of cancer cells 101. Inhibited palmitoylation results in PD-L1 lysosomal degradation, blockade of PD-L1/PD-1 interaction, activation of T-cell immune responses against tumors 101. Interestingly, the palmitoylation of FASN itself is also enhanced notably in cisplatin-resistant bladder cancer cells and 2-BP can suppress FASN expression in a dose-dependent manner potentially via inhibiting its palmitoylation 49.

TEAD transcription factors have also been implicated as oncogenic factors and involved in cell fate determination, polarity, proliferation, and survival 102. They combine with the co-activator YAP/TAZ to regulate the signal transduction of Hippo pathway 103 **(Figure 3b)**. TEADs undergo autopalmitoylation independent of PATs through insertion of palmitate into a conserved hydrophobic pocket 41. It is essential for TEAD's binding to YAP/TAZ, moreover, it can happen even under physiological concentrations of palmitoyl-CoA (0.1-10 μM) 41. Another research shows the palmitoylation of TEADs is regulated by palmitate synthases (ACC and FASN) and depalmitoylases (APT2 and ABHD17A) in response to cell density 57. Thus, the hyper-palmitoylation in TEAD-YAP/TAZ may be one of the mechanisms underlying the overexpressed FASN and activated lipid metabolism in many cancers. Recently, DC-TEADin02 has been identified as a potent, selective, covalent TEAD autopalmitoylation inhibitor with the IC50 value of 197 ± 19 nM for TEADs transcription activity 104. Holden et al. 105 discovers that a small molecule that binds to TEAD lipid pocket can change TEAD palmitoylation and transform it into a dominant-negative transcriptional repressor, causing tumor growth inhibition in a xenograft model. Thus, it is promising to target FA synthesis and TEADs palmitoylation for cancer therapy.

The melanocortin-1 receptor (MC1R), a melanocyte-specific GPCR, plays a crucial role in pigmentation. Palmitoylation of MC1R at Cys^315^ is required for MC1R tumor suppressor function 106. Targeting APT2 (using ML349) and increasing MC1R palmitoylation, therefore, represses UVB-induced melanomagenesis and reduce melanoma risk, especially in individuals with red hair 107. Another classic example is the RAS family of small GTPases. Palmitoylation of H-Ras, N-Ras, and K-Ras-4A controls the distribution and function of these proteins. For instance, palmitoylation of N-Ras plays a pivotal role in the development of melanoma and hematopoietic malignancies. Mice transplanted with bone marrow cells expressing an activated but non-palmitoylatable mutant (NRAS^G12D, C181S^) stay alive for 2 years, whereas mice transplanted with NRAS^G12D^ cell die of leukemogenesis within 3 months 44. Recently, a study found artemisinin could bind covalently and inhibit ZDHHC6 to suppress N-Ras palmitoylation, affect N-Ras subcellular localization, and disrupt the oncogenic signaling cascades 108.

## Palmitoylation in inflammation and infection in response to fatty acids

Accumulating evidences have revealed the inextricable link between lipid metabolites and immune response under physiological and pathological conditions 109. HFD-induced obesity has been considered as a contributing factor to morbidity and mortality from sepsis 110. A key output for these consequences involves palmitoylation of proteins by metabolites. FA synthesis is associated with activated immune cells and essential for the synthesis of some proinflammatory mediators. Moreover, palmitate can modify key proteins via palmitoylation and alter their functions.

Overexpression of ACSL1 exacerbates the detrimental effects of FAs on inflammatory activation, while knock-out of ACSL1 in myeloid cells suppresses NLRP3 inflammasome activation and the production of IL-1β 111. As described above, ACSLs are positively associated with the palmitoylation of many proteins, thus palmitoylation is one potential mechanism involved in ACSL signaling. CD36 is also involved in the induction of inflammation through the assembly of Toll-like receptor (TLR) TLR4, TLR6 and activation of NLRP3 inflammasome, the downstream effectors including NF-κB, JNK pathways, ER stress and release of other proinflammatory cytokines 112, 113. A HFD promotes the palmitoylation of CD36 in mice liver, leading to increased levels of cytokines/chemokine (e.g. TNFα, IL-6) and fibrosis markers (e.g. TGFβ) 62. Inhibition of CD36 palmitoylation can suppress JNK signaling pathway and reduce the CD36/Lyn/Fyn complex** (Figure 3a)**, representing a potential therapeutic strategy for suppressing CD36-mediated inflammatory signaling 62.

*De novo* palmitate synthesis by FASN is an indispensable regulator of inflammatory and immune response. Study has shown that FA-induced palmitoylation may regulate inflammatory reaction, particularly TLR/MYD88 signaling 47. MYD88, a common adapter molecule in the TLR family, is FASN-dependent palmitoylated. A combination of inhibiting FASN and depleting exogenous FAs blocks MYD88 palmitoylation and TLR signaling, indicating that the endogenous FA synthesis and CD36-mediated exogenous FA uptake are two important sources for MYD88 palmitoylation, while the palmitoylation is required for downstream IRAK4 recruitment and subsequent TLR activation 47. In contrast, inhibition of MYD88 palmitoylation improves the chemotactic activity of neutrophils and potentially reduces mortality from sepsis **(Figure 3c)**. A proteomics identifies TLR 2, 5, 10, CD80, CD86 can be palmitoylated in dendritic cells 114. S-palmitoylation of TLR2 is required for its trafficking to the cell surface and interaction of its ligands, while the palmitoylation-deficient mutant of TLR2 impairs the activity of NF-κB and production of inflammatory cytokines in response to pathogens 114.

Recently, Signal transducer and activator of transcription 3 (STAT3), the key T_H_17 cell differentiation stimulator 115, was found palmitoylated on cysteine 108 by ZDHHC7. Disruption of either palmitoylation or depalmitoylation negatively affects T_H_17 cell differentiation, and thus represents a potential therapeutic strategy for the treatment of inflammatory bowel diseases 116. Given the importance of STAT3 in cancer, STAT3 palmitoylation is likely to play a significant role in cancer too. On the other hand, Berod et al. 117 have demonstrated the ACC inhibitor that blocks *de novo* FA synthesis may attenuate T helper 17 (T_H_17) cell-mediated autoimmune disease. However, it is worth noting that the effect of ACC inhibitor is not mediated by inhibition of protein palmitoylation 117. To be specific, none of the protein palmitoylation levels are changed upon ACC inhibitor, which includes the palmitoylation levels of T cells differentiated under T_H_17 conditions, and the palmitoylation levels of STAT3, AMPK and FYN 118. Therefore, we have to admit that the relationship between FA synthesis and palmitoylation levels remains uncertain and depends on different scenarios.

Lipopolysaccharide (LPS) stimulates strong pro-inflammatory response of immune cells partly through palmitoylation 119. Immunity helps the body combat infection, whereas exaggerated reaction to LPS leads to an uncontrollable sepsis. A palmitoylome of in RAW264 macrophage-like cells reveals that LPS induces evident alteration in the abundance of palmitoylated proteins 120. LPS promotes the palmitoylation and activation of type II phosphatidylinositol 4-kinase (PI4KII) β, which can phosphorylate phosphatidylinositol and control PI(4,5)P2 synthesis, resulting in the synthesis of pro-inflammatory cytokines 120.

Palmitoylation of stimulator of interferon genes (STING) at the Golgi is essential for the activation of STING and innate immune response 121. STING activates TBK1 and then IRF3, resulting in the production of type I IFNs. It has also been demonstrated that dietary palmitate induces STING activation and pro-inflammatory cytokines in hepatic steatosis 122. Inhibition of STING palmitoylation with small molecule compounds abolishes the type I IFN response and attenuates pathological features of auto-inflammatory disease in mice 123. TNF-R1 also undergoes palmitoylation, and mutation of the palmitoylated site impairs TNF-R1 localization and downstream signaling 124. NOD1 and NOD2 are palmitoylated in primary monocytes and macrophages and the palmitoylation is required for membrane localization and downstream NF-kB, MAPK activation in Salmonella typhimurium infection model 125. All of these palmitoylated proteins are essential for immune response and signal transduction.

In terms of virus infections, an inhibitor of FASN (TVB-3166) reduces the replication of respiratory syncytial virus progeny *in vivo* and *in vitro* 126. The conserved RNA capping enzyme nsP1 from alphavirus can be palmitoylated and mutations that prevent nsP1 palmitoylation reduce virus replication 127. Another alphavirus, i.e., Chikungunya Virus infection is also dependent on FASN to produce PA for the palmitoylation of nsP1 128. C75, the FASN inhibitor, reduces nsP1 palmitoylation and plasma membrane localization while PA can restore the palmitoylation and enhance virus replication. FASN contributes to the replication of other viruses, like hepatitis C virus 129, HIV 130, dengue virus 131, and rotavirus 132. And palmitoylation of viral proteins promotes binding to membranes, virus replication, entry into host cells and release of viral particles 133-135. It is intriguing whether enhanced FASN palmitoylation could be a missing link in the molecular mechanism of virus infection and serve as a promising therapeutic target.

## Palmitoylation in neurological and psychiatric disorders

Palmitoylation is enriched in the nervous system and may play critical roles in regulating synaptic function and neuronal protein localization 136. Disruption of palmitoylation has been implicated in the pathogenesis of neurodegenerative diseases and psychiatric disorders, such as Alzheimer's disease (AD) 137, Huntington disease (HD) 138, 139, Parkinson's disease (PD) 140 and schizophrenia 141, depression 142. To be specific, 41% of synaptic genes (1838 human genes in total) encode a palmitoylated protein from the datasets of 15 palmitoylation proteomics studies 143. This significant enrichment of palmitoylated proteins indicates that palmitoylation is a pivotal regulator of the synapse. The associated diseases of those genes include cancers, Schizophrenia, HD and other disorders of the nervous system 143. Interestingly, dysregulation of palmitoylation leads to cell death or cell over-proliferation depending on the proteins involved. For instance, loss of function of DHHC17 is involved in HD while hyper-activation of DHHC17 may cause cancer 144, 145. The molecular mechanism underlying the impact of palmitoylation on neurological disorders is still not elucidated.

AD is characterized by neurotoxic extracellular β-amyloid (Aβ) aggregation and formation of amyloid plaques in brain 146. Aβ production is closely associated with amyloid precursor protein (APP) and processed by β-secretase (BACE1), while both APP and BACE1 can be palmitoylated. The hyper-palmitoylation of APP meditated by DHHC7 and DHHC21 activates amyloidogenic pathway and promotes Aβ aggregation 137, while BACE1 palmitoylation could also increase Aβ production 147. Emerging studies have exemplified the essential role obesity and insulin resistance play in AD pathogenesis. Obese patients even exhibit defective learning and memory ability like AD patients 148. However, some declare that there is no association between them except that diabetes can be a risk factor for vascular dementia 149.

In PD, α-synuclein (α-Syn) accumulates in insoluble inclusions. Inhibitors of stearoyl-CoA desaturase robustly prevent the α-Syn inclusions and reduce α-Syn neurotoxicity 150. Accordingly, conditioning cells in SFAs rescued, whereas unsaturated FAs worsened, the α-Syn phenotypes 150. Interestingly, Ho et al. 151 have demonstrated α-Syn pathophysiology is associated with APT1-regulated palmitoylation. Since ATP1 is an important depalmitoylase, inhibiting ATP1 corrects a α-Syn-dependent MAP6 palmitoylation deficit, ameliorates α-Syn cytoplasmic inclusions and α-Syn-mediated neurotoxicity 151. Therefore, upregulating palmitoylation represents a novel therapeutic strategy for synucleinopathies like PD. Moreover, in PD models of Drosophila expressing Pink and Parkin mutant, supplementing dietary stearic acid (C18:0) can increase TFR1 acylation, and thus rescue the mitochondrial dysfunction and PD phenotypes caused by genetic defects 58, which identifies FA as a key regulator of mitochondrial function via acylation in neurodegeneration.

HD is caused by the huntingtin gene mutation that encodes an abnormal huntingtin protein (HTT). Huntingtin protein is also palmitoylated by DHHC17 and DHHC13. In HD patients, interaction between HTT and its PATs is damaged due to the mutation, leading to a distinct decline in HTT palmitoylation 152. In addition, the activity of DHHC17 appears to be compromised in HD, and the palmitoylation levels of other DHHC17's substrates are also affected, which leads to neuronal toxicity 153. In a recent study, ML348 (the inhibitor of APT1) could efficiently cross the blood-brain barrier to restore palmitoylation levels and reverse neuropathology in HD mice, and thus hold therapeutic interest for HD 154.

HFD-induced palmitate deposition in the hippocampus increases palmitoylation of AMPA glutamate receptor subunit GluA1, leading to reduced GluA1 phosphorylation, suppressed AMPAR response and impairment of cognitive function 60** (Figure 3d)**. Palmitate and insulin are used *in vitro* to mimic the HFD-induced insulin resistance and metabolic changes. When using oleic acid as a substitute for palmitate, there is no detection of increased GluA1 palmitoylation, which again illustrates the critical role palmitate plays. Moreover, as the major enzyme of GluA1 palmitoylation, ZDHHC3 activity is positively regulated by its autopalmitoylation level, while HFD induces ZDHHC3 overexpression and activation 60. In contrast, HFD feeding does not appear to affect ZDHHC4 or ZDHHC5 expression and activity in adipose tissue 63, indicating HFD may have varying effects on ZDHHCs and ZDHHCs play different roles in different tissues. Interestingly, in another research, maternal HFD exposure also enhances GluA1 palmitoylation and affects adult male offspring hippocampal synaptic plasticity and cognitive performance multi-generationally 155. Moreover, 2-BP diminishes GluA1 palmitoylation and restores cognitive function in HFD mice as well as their offspring 60, 155.

Another palmitoylated protein which plays a pivotal role in developmental synaptic plasticity and learning cognitive function is PSD-95 156. Palmitoylation is required for PSD-95 to regulate synaptic trafficking of AMPARs and activity-dependent plasticity. Overexpression of ABHD17B selectively depalmitoylates PSD-95 and decreases the synaptic clustering of PSD-95 and GluA1 36. In addition, high anxiety is accompanied by inhibited gephyrin palmitoylation and suppressed synaptic function of GABA_A_R in the basolateral amygdala of rats, while diazepam mediates the anxiolytic effect through activating DHHC12 and increasing gephyrin palmitoylation 157.

Serotonin 1A receptor (5-HT1AR) is palmitoylated by DHHC21 in brains and involved in major depressive disorder. Inhibited palmitoylation of 5-HT1AR can significantly affect the signal transduction and lead to depression-like behavior, and this inhibition of palmitoylation is also found in people who suffered depression and committed suicide 142. The palmitoylation of D2 dopamine receptor, a G protein-coupled receptor, is required for the stability and trafficking of receptors, and the aberrant palmitoylation results in dopamine dysregulation syndrome, linked to schizophrenia and PD as well 158.

It is possible that palmitate in the nervous system may alter the function of numerous synaptic proteins through palmitoylating them, resulting in either inhibitory (e.g. GluA1 or GluA2) or enhanced synaptic plasticity (e.g. GABAA receptor γ2 or PSD95) 60. The scenario may be more complex when palmitoylation of different cysteine residues in the same target produces opposite effects (e.g. NR2A or NR2B) 159. The crosstalk between metabolism and neurological dysfunction mediated by aberrant palmitoylation remains to be explored in the future.

## Future perspectives

Lipid metabolites play an essential role in the regulation of physiological and pathologic responses. Although palmitate synthesis or metabolism represents a limiting step for palmitoylation, interplay between these two processes is only rudimentary understood. Emerging evidence indicates that palmitoylation can be modulated by lipid signals and metabolic activities, and in return palmitoylation affects protein function and stability. Moreover, aberrant palmitoylation has been linked to many human diseases including cancers, metabolic disorders, inflammation and neurodegenerative diseases. Targeting palmitoylation could be an attractive therapeutic strategy.

The dynamic palmitoylation-depalmitoylation cycles are crucial to cell signal transduction. However, there remain many technical hurdles to the study of palmitoylation. The absence of a specific sequence motif in palmitoylated protein makes it difficult to predict and determine the exact sites. The lack of commercial antibodies and high hydrophobicity also need to be conquered. Fortunately, the unstable nature of the thioester linkage enables the detection of palmitoylation by acyl-biotin exchange, which is a convenient assay to use. Recent bioinformatics and proteomics are employed to predict and identify increasing unknown palmitoylated proteins, and new method like click chemistry is used to investigate this dynamic process where cells are metabolically labeled with a palmitic acid analog containing an alkyne group. The exogenous chemical reporters (e.g., fluorescent proteins) can be taken up and traced in cell cultures to imitate the *in vivo* status. Development of new probes like palmitoylated state-specific antibodies will be of great research significance.

There remain many unanswered questions about lipid metabolism and palmitoylation. First, we need to identify whether abnormal lipid levels (e.g., palmitoyl-CoA level) are linked to deregulated protein palmitoylation. Particularly in cancers, could high-saturated fat diets promote tumor progression through enhancing the palmitoylation of certain oncoproteins? If so, how do dietary intake and cellular lipid metabolism affect palmitoylation and how could we avoid the unfavorable outcomes? In contrast, if a high-fat diet inhibits the palmitoylation of a certain protein, what is the potential mechanism? This is in urgent need of in-depth investigation. In view of recent evidences, it is most likely that palmitate metabolites exerted different effects on different proteins. Also, how the alterations in metabolite levels are initiated and transmitting signals in cascade, and to what extent the observations in animal models are similar to humans, needs to be determined. Such studies may provide insights into the underlying mechanism of lipid-related diseases. Meanwhile, the physiological functions of ZDHHCs have not been studied thoroughly, and the enzyme-substrate pairs have not been established yet. Given that mutations of ZDHHC proteins are observed in various human diseases, can we develop specific inhibitors for individual PATs or APT/PPT enzymes? Such inhibitors may selectively suppress the activities of some difficult-to-drug oncoproteins. On the other hand, FASN and ACSL are important for endogenous palmitate production, and CD36 serves as the major player for cell palmitate uptake. The inhibitors of them affect the palmitoylation of some proteins, thus, targeting the bioavailability of palmitate is another potential tactic to regulate palmitoylation. Preclinical studies have shown that some common chemical inhibitors that have been developed over the past years could regulate palmitoylation through controlling the relevant enzymes or the lipid substrate production** (Table 1)**. Whether these inhibitors have clinical application value remains to be explored.

The idea that lipid metabolites may directly or indirectly change protein modification, as well as affect both activation and repression signals, opens up new opportunities to study novel mechanisms underlying the interaction between metabolism and human diseases. Future studies on drugs that modulate palmitoylation might provide novel therapeutic strategy.

## Figures and Tables

**Table 1:** Modulation of palmitoylation as a potential therapeutic strategy

Chemical name	Mechanism	Example of palmitoylated protein and relevant outcome		References
2-BP	A non-specific, irreversible inhibitor of PATs	Inhibit GluA1 palmitoylation	Restore cognitive function in high-fat diet mice	60
Cerulenin	An irreversible inhibitor of FASN and PATs	Inhibit XBP1 palmitoylation	Induce cell death in Glioblastoma multiforme	160
Palmostatin B	An inhibitor of APT1/APT2/ABHD17	Ras palmitoylation	Induce reversion in oncogenic; HRasG12V-transformed fibroblasts	161
ML348	A selective reversible inhibitor of APT1	CD36 palmitoylation	Eliminate fatty acid uptake activity of CD36	64
ML349	A selective reversible inhibitor of APT2	Scrib palmitoylation	Rescue Scrib tumor suppressor properties	162
Orlistat	An FDA-approved anti-obesity drug that inhibits FASN	Inhibit EGFR palmitoylation	Reduce tumor growth in EGFR mutated non-small cell lung cancer	50
C75	An inhibitor of FASN	Inhibit nsP1 palmitoylation	Reduce Chikungunya virus replication	128
TVB-3166/TVB-3664	An inhibitor of FASN	Inhibit tubulin palmitoylation	Inhibit xenograft tumor growth	163
Triacsin C	An inhibitor of acyl-CoA synthetases	Inhibit eNOS palmitoylation	Enhance NO dependent vascular smooth muscle relaxation	53
SSO	An inhibitor of CD36	Slightly reduce MYD88 palmitoylation	Increase the survival of mice with sepsis	47

Abbreviation: 2BP: 2-Bromopalmitate; PATs: palmitoyl acyltransferases; GluA1: AMPA receptor subunit 1; FASN: fatty acid synthase; XBP1: X-box binding protein 1; APT: acyl-protein thioesterases; ABHD: the α/β hydrolase domain proteins; Scrib: scaffolding protein Scribble; EGFR: epidermal growth factor receptor; eNOS: endothelial nitric oxide synthase; SSO: sulfo-N-succinimidyl oleate; MYD88: myeloid differentiation primary response protein.

## Abbreviations

DNL: *de novo* lipogenesis
PAT: palmitoyl acyltransferases
APT: acyl-protein thioesterases
ABHD: the α/β hydrolase domain protein
TEAD: Transcriptional enhanced associate domain
GPCR: G protein-coupled receptor
FASN: fatty acid synthase
ACS: Acyl-CoA synthetases
ACC: Acetyl-CoA carboxylase
NASH: non-alcoholic steatohepatitis
eNOS: endothelial nitric oxide synthase
LMO4: LIM domain only 4
PD-L1: programmed death receptor ligand
EGFR: Epidermal growth factor receptor
2-BP: Bromopalmitate
MC1R: The melanocortin-1 receptor
STING: stimulator of interferon genes
TLR: Toll-like receptor
PI4KII: type II phosphatidylinositol 4-kinase
MYD88: myeloid differentiation primary response protein
LPS: Lipopolysaccharide
STAT3: Signal transducer and activator of transcription 3
AD: Alzheimer's disease
HD: Huntington disease
PD: Parkinson's disease
APP: amyloid precursor protein
5-HT1AR: Serotonin 1A receptor
